# Primary Arthroscopic Repair of the Anterior Cruciate Ligament with Femoral Fixation Using an Adjustable EndoButton and Dynamic Reinforcement with High-Strength Suture

**DOI:** 10.1055/s-0045-1814430

**Published:** 2026-03-24

**Authors:** Antonio Carlos Moscon, Fabrício Luz Cardoso, Daniel Rocha de Almeida Braga, Claudio Gattás

**Affiliations:** 1 Knee Surgery Group, Clínica Ortocity – Orthopedics, Fractures, and Rehabilitation, São Paulo, SP, Brazil

**Keywords:** anterior cruciate ligament, arthroscopy, suture techniques, artroscopia, ligamento cruzado anterior, técnicas de sutura

## Abstract

Primary anterior cruciate ligament (ACL) repair has reemerged as an alternative in selected cases thanks to advances in arthroscopic techniques and the development of modern fixation devices. The present paper describes the arthroscopic ACL repair technique with femoral fixation using an adjustable EndoButton (Smith & Nephew) combined with dynamic reinforcement using a high-strength suture. This technique is primarily indicated for acute proximal ruptures (Sherman et al. types I and II), which present a good ligament remnant and healing potential. The procedure aims to preserve the original anatomy and proprioception, reduce the morbidity associated with graft harvesting, and maintain future reconstruction options. The technique involves suturing the ligament stump, using adjustable femoral fixation, and applying internal bracing to provide additional biomechanical stability. The proposed postoperative protocol, based on the rationale of stability, prioritizes early mobilization, progressive weight-bearing, and structured rehabilitation. This approach represents a promising alternative for carefully-selected patients, especially young athletes and skeletally-immature subjects, provided the indications and limitations of the technique are respected. Long-term studies are needed to consolidate the clinical efficacy of this technique.

## Resumo

O reparo primário do ligamento cruzado anterior (LCA) tem ressurgido como alternativa em casos selecionados, impulsionado pelo avanço das técnicas artroscópicas e pelo desenvolvimento de dispositivos modernos de fixação. Este trabalho descreve a técnica de reparo artroscópico do LCA com fixação femoral em EndoButton (Smith & Nephew) ajustável associada ao reforço dinâmico com sutura de alta resistência. A indicação mais favorável ocorre em rupturas proximais agudas (tipos I e II de Sherman et al.), nas quais há bom remanescente ligamentar e potencial de cicatrização. O procedimento visa preservar a anatomia e a propriocepção originais, reduzir a morbidade da retirada de enxertos e manter opções futuras de reconstrução. A técnica inclui sutura do coto ligamentar, fixação femoral ajustável e reforço interno, o que proporciona estabilidade biomecânica adicional. O protocolo pós-operatório proposto, baseado no fundamento lógico da estabilidade, privilegia mobilização precoce, carga progressiva e reabilitação estruturada. Esta abordagem representa uma alternativa promissora para pacientes criteriosamente selecionados, sobretudo atletas jovens e indivíduos em crescimento, desde que respeitadas as indicações e as limitações da técnica, sendo necessária a validação por estudos de longo prazo para a consolidação de sua eficácia clínica.

## Introduction

Over the past two decades, advances in arthroscopic techniques and clinical evidence of reliable anterior cruciate ligament (ACL) healing in selected cases, with return-to-sport rates comparable to those of ACL reconstruction (ACLR), sparked an interest in the primary repair of this ligament. 1 2 3

The rationale for primary ACL repair relies on the potential benefits of preserving proprioception, maintaining the original ligament anatomy, and minimizing bone damage with the creation of smaller or fewer tunnels. This strategy also aims to eliminate complications associated with the harvesting of autologous grafts, such as anterior knee pain, hamstring or quadriceps weakness, cramps, and the risk of rupture of the remaining tendons. 2 4

Technological improvements, with the emergence of high-strength sutures and adjustable suspension systems, have enabled the combination of anatomical repair with internal reinforcement techniques (augmentation). This combination aims to promote biological healing, enhance biomechanical safety, and contribute to an expectation of earlier functional recovery, with increased range of motion and a more natural subjective knee sensation. 5 6

The Sherman et al. 7 classification, which identifies which proximal ACL injuries are more prone to healing, was a fundamental milestone in ACLR research.

Recent studies 1 6 report rerupture rates ranging from 7 to 20% in proximal lesions (Sherman et al. 7 types 1 and 2). Currently, these injuries are the most investigated. Although these data support primary repair as a viable alternative in specific scenarios, long-term follow-up studies are still required for a definitive validation of the technique.

The current article provides a detailed description of the arthroscopic technique for primary ACLR with femoral fixation using an adjustable EndoButton (Smith & Nephew) and dynamic reinforcement with high-strength suture. Documentation and refinement of procedural details used a fresh-frozen cadaver model to facilitate the step-by-step demonstration. The study presents the proposed postoperative care and discusses the biomechanical rationale behind this technique, highlighting its theoretical advantages over conventional methods employing external implants.

## Patient selection

The success of ACLR depends on meticulous patient selection. The initial evaluation must include detailed medical history, complete physical examination, and analysis of imaging scans.

Patients with ACL rupture frequently report a sharp popping sound at the time of the injury, followed by hemarthrosis and subjective knee instability resulting from anterior tibial translation unresisted by the ruptured ligament. 8

Whenever possible, the physical examination must assess ligamentous laxity using the Lachman and pivot shift tests, which have high specificity to detect anterior tibial and rotational instability. Plain radiographs can help identify associated fractures, such as Segond's fracture, while magnetic resonance imaging (MRI) is essential to confirm the diagnosis, assess the remaining ligament, and detect concomitant injuries.

The ideal indications include acute proximal ruptures, particularly Sherman et al. 7 type-I femoral avulsions, with a good ligament remnant and a short interval between injury and repair, factors that favor greater healing potential and satisfactory clinical outcomes. 6 8 9 Pediatric or skeletally-immature patients also benefit from ACL biological preservation, provided that the integrity of the ligament stump is preserved. 9

In contrast, chronic injuries, ruptures in the mid-substance or distal third of the ligament, degenerated tissue, and recurrences in previously-repaired ligaments represent contraindications, as they compromise the biomechanical quality of the repair and increase the risk of failure. 9

Even in cases of suspected proximal rupture on imaging, the definitive decision between primary repair and reconstruction is made intraoperatively, after direct assessment of the type of rupture and the tissue quality. Moreover, a previous discussion of graft alternatives must occur if reconstruction is required.

Among special populations, such as young athletes in pivoting sports and adults with high physical demands, primary repair may be a viable alternative in selected situations. However, the literature 10 recommends careful evaluation, prudent indication, and structured rehabilitation, given the increased risk of rerupture in these groups.

## Description of the technique

### Positioning

The patient is in supine position, under general or spinal anesthesia, with a pneumatic tourniquet on the proximal third of the thigh. The limb for treatment is placed on a lateral support for full flexion and extension. Antibiotic prophylaxis is administered according to the institutional protocol.

### Arthroscopic portals and inspection

The portals include an anterolateral (optical), an anteromedial (working), and an additional anteromedial portal to construct the femoral tunnel. The joint undergoes inspection and treatment of any associated meniscal or chondral lesions.

### ACL identification and stump preparation

**Fig. 1:**
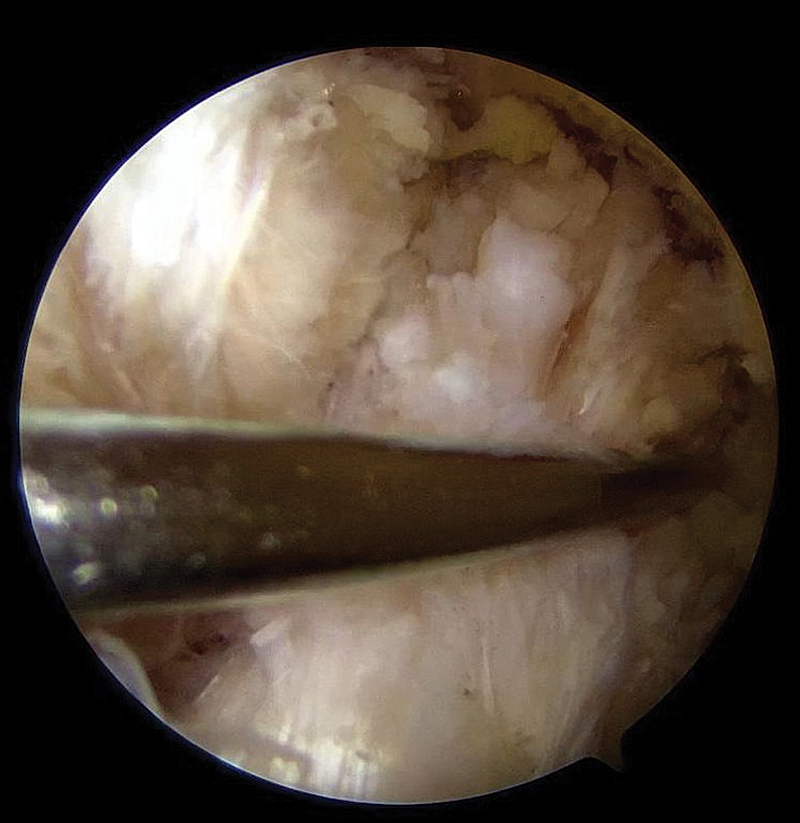
Identification of the proximal anterior cruciate ligament (ACL) injury.

The proximal ACL tear is identified, preserving as much of the remaining ligament as possible ( Fig. 1 ). Debridement is minimal, using a shaver or radiofrequency only to remove unstable fibers, preserving vascularization. The femoral footprint is subtly roughened with a shaver or curette to promote healing.

### Stump suture

**Fig. 2:**
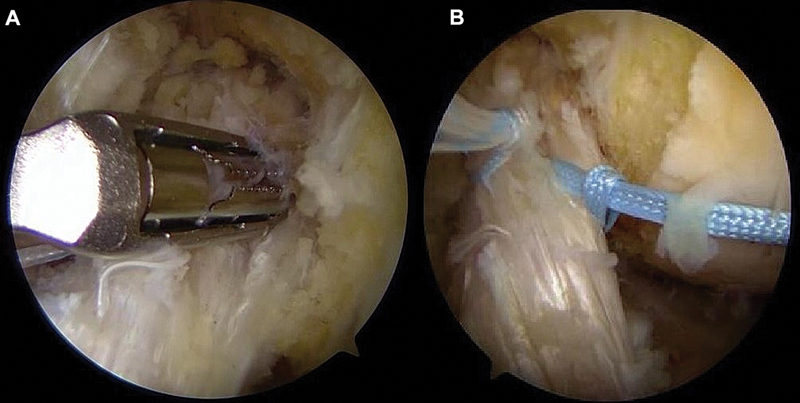
Positioning of the arthroscopic suture passer ( **A** ). Performance of two high-strength sutures involving the anteromedial and posterolateral bundles ( **B** ).

Using an arthroscopic suture passer, 2 sutures are performed with high-strength (no. 2) 100% polyethylene threads in a lasso-loop or locked configuration, from distal to proximal, involving the anteromedial and posterolateral bundles ( Fig. 2 ). The sutures remain free for subsequent tensioning.

### Femoral fixation

**Fig. 3:**
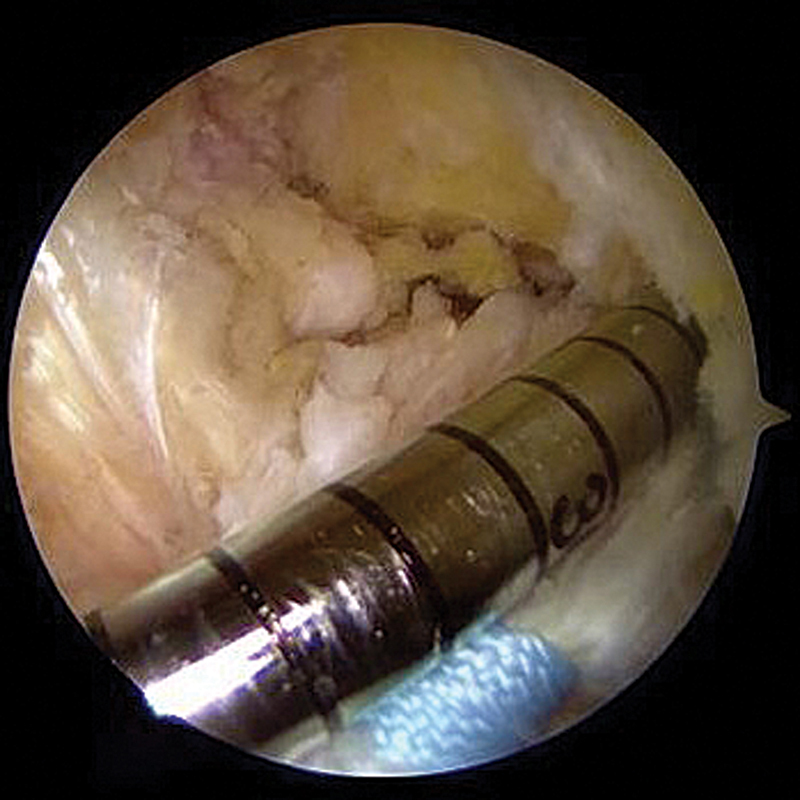
Construction of the femoral tunnel in the anatomical ACL footprint via the anteromedial accessory portal using a 4.5-mm drill bit.

**Fig. 4:**
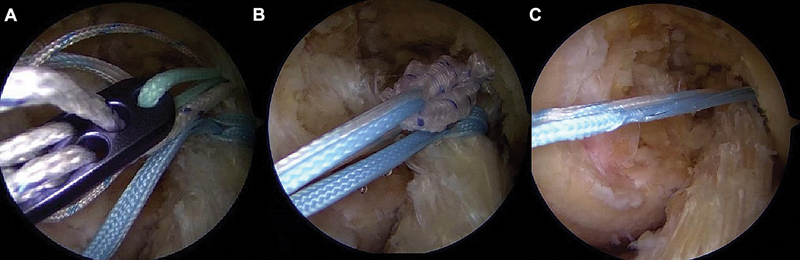
Passing the suture threads through the adjustable loop of the EndoButton ( **A** ). Guiding the ACL stump through the femoral tunnel ( **B** ). Suspension fixation of the repaired ACL to the medial wall of the lateral femoral condyle ( **C** ).

With a femoral guide wire and a 4.5-mm drill bit, a tunnel is created in the anatomical footprint of the ACL through the anteromedial accessory portal, with the knee under hyperflexion ( Fig. 3 ). The ligament stump sutures are passed through the adjustable loop of the EndoButton, guided through the tunnel, and fixed in cortical suspension. The ligament stump is reduced to its footprint by pulling the sutures passed “astride” through the adjustable loop ( Fig. 4 ).

### Reinforcement (internal brace)

The same sutures used to close the umbilical stump act as internal reinforcement, providing additional support during ligament healing ( Fig. 4 ).

### Tibial fixation and final adjustment

**Video 1** Step-by-step of the primary ACL suture technique.


**Fig. 5:**
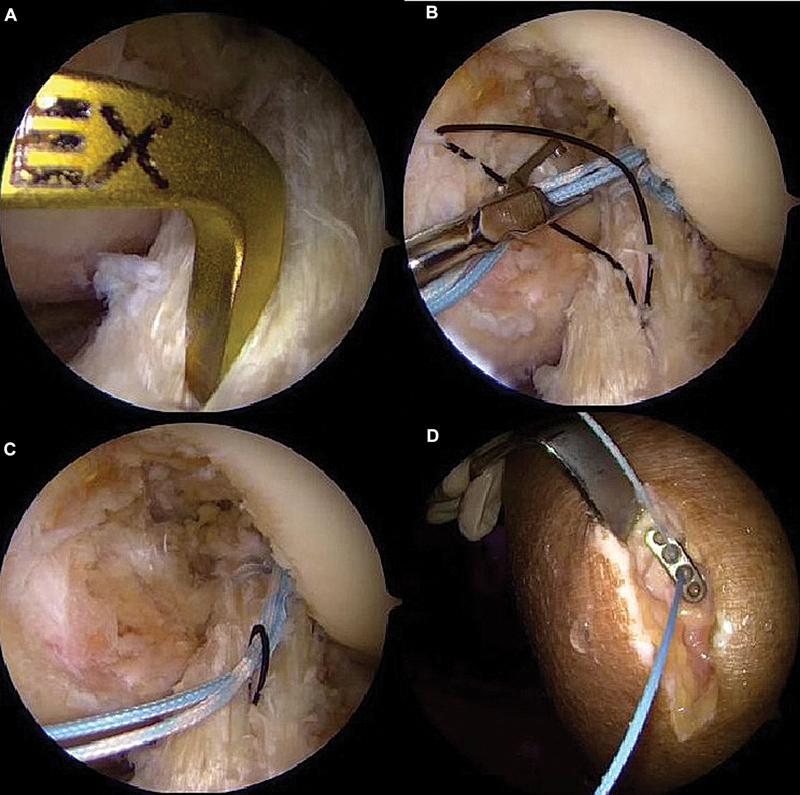
Positioning of the tibial tunnel ( **A** ). Passing the wires through the distal ACL ( **B** ). Guiding the internal brace wires into the tibial tunnel ( **C** ). Fixation to the anterior tibial cortex with EndoButton ( **D** ).

The wires are guided through a 4.5-mm tibial tunnel to the anteromedial band insertion point and secured to the anterior tibial cortex with an EndoButton or knotless anchor ( Fig. 5 ). Knee flexion in 0 to 20° enables the proper tensioning of the adjustable loop.

A follow-up arthroscopy verifies isometry, absence of impingement, and stability after the repair. Next, three to four microfractures are performed on the medial aspect of the lateral femoral condyle, adjacent to the footprint, to promote biological (bone marrow) stimulation. 11 Drilling into the subchondral bone releases mesenchymal stem cells and bone-marrow growth factors at the repair site, which, by analogy to studies 11 using bone marrow aspirate concentrates (BMACs) in ligament reconstructions, seeks to accelerate and optimize the healing and maturation of the repaired ligament tissue. Video 1 shows the entire procedure, demonstrating the technique step by step.

### Closure

After thorough irrigation, the portals are closed with simple nylon sutures, followed by the application of a compression dressing.

### Postoperative care

The postoperative protocol involves early mobilization, progressive weight bearing as tolerated, and the initial use of an articulated orthosis. The goal is to stimulate biological healing without compromising joint stability.

## Conclusion

This technique combines the principles of primary anatomical ACLR with the use of modern suspension devices, incorporating internal reinforcement through ligament stump sutures passed “astride” the EndoButton loop.

This configuration provides dynamic fixation, promoting continuous approximation of the ligament stump to the femoral footprint even in the presence of slight tibial anteriorization. The choice of an adjustable EndoButton, instead of “fixed” fixations, enables the ideal tensioning and in-situ adjustment of the repair after cortical fixation. This continuous adjustment capability is fundamental for a dynamically-reinforced repair, as it confers a biomechanical advantage to the system, preventing tension loss that can occur during joint movement cycles and favoring constant coaptation of the stump to the biological bed.

This technique is an alternative with key theoretical and biomechanical potential, as it spares native tissue and maintains the possibility of future surgical revisions with lower procedural complexity.

Although the current paper is a “technical note”, limited to a detailed description of the procedure, we used this technique in 15 patients during our initial experience. However, there are not enough results for analysis. The clinical validation of its benefits requires proper follow-up. As such, we plan to prospectively follow up the patients for 6 months to 2 years, through clinical and functional reassessments, using validated knee scores (such as that of the International Knee Documentation Committee) and MRI evaluations at 3, 6, and 12 months postoperatively, aiming to consolidate the results and compare the efficacy with that of the gold standard.
